# Associations between delayed completion of high school and educational attainment and symptom levels of anxiety and depression in adulthood

**DOI:** 10.1186/s12888-016-0765-1

**Published:** 2016-03-15

**Authors:** Ole Melkevik, Lars Johan Hauge, Pernille Bendtsen, Anne Reneflot, Arnstein Mykletun, Leif Edvard Aarø

**Affiliations:** National Research Centre for the Working Environment, Copenhagen, Denmark; Norwegian Institute of Public health, Oslo, Norway; National Institute of Public Health, University of Southern Denmark, Copenhagen, Denmark; Department of Community Medicine, University of Tromsø, Tromsø, Norway; Center for Work and Mental Health, Nordland Hospital Trust, Bodø, Norway; University of New South Wales, School of Psychiatry, Sydney, Australia

## Abstract

**Background:**

There is a higher prevalence of anxiety and depression among adults with lower educational attainment. Delayed completion of high school (HS) is common and represents a potentially complicating factor in the relationship between educational attainment and anxiety and depression. This study aims to investigate whether delayed HS completion is associated with symptom levels of anxiety and depression in adulthood and whether it interacts with later educational attainment in predicting symptom-levels of anxiety and depression in adulthood.

**Methods:**

The sample consisted of 10 149 participants from the Nord-Trøndelag Health Survey (HUNT 3) between 30 and 46 years of age in 2006. The outcome variables were symptoms of anxiety and depression as measured by the HADS scale. Variables measuring educational attainment were obtained from the National Educational Database in Norway. We used linear regression to estimate associations between educational attainment, delayed HS completion and symptom levels of anxiety and depression in adulthood.

**Results:**

We found delayed HS completion to be associated with higher symptom levels of both anxiety and depression. There was a dose–response association suggesting that each additional year of delay in HS was associated with higher symptom levels for both anxiety and depression. Mean symptom levels of both anxiety and depression were significantly lower among individuals who completed HS within a normative timeframe vs those who were substantially delayed in their HS completion. For anxiety symptoms, we found a statistically significant interaction between delayed HS completion and later educational attainment. This interaction suggested that individuals with a combination of being delayed in HS and having no higher educational attainment had significantly higher levels of anxiety symptoms than all other combinations of later educational attainment and normative/delayed HS completion. For depression, associations between predictors and symptom levels were additive.

**Conclusions:**

Delayed HS completion is associated with symptom levels of both depression and anxiety and interacts with later educational attainment in predicting symptom levels of anxiety. Individuals with a combination of delayed HS completion and lower educational attainment had particularly high symptom levels of anxiety.

## Background

Several studies indicate that there is a higher prevalence of anxiety and depression among adults with low educational attainment [1–5]. Two major theories have been proposed to explain this pattern. Social causation theory suggests that there is an elevated risk among individuals of low SES due to mechanisms such as increased stress [6], limited coping recourses [7], or lack of occupational direction, control and planning [8]. Social selection theory on the other hand explains the educational gradient in anxiety and depression by impaired social mobility among individuals with mental disorders [9], suggesting that early onset of anxiety and/or depression impairs individuals’ ability to attain higher educational credentials.

The processes of selection and causation are not necessarily mutually exclusive and have been found to vary across types of disorders [10]. Nevertheless, a number of studies argue that social causation mechanisms are more important in explaining the distribution of anxiety and depression in the adult population [9–13]. Results from other studies provide evidence in support of social selection theory as they suggest that individuals with early onset internalizing disorders are less likely to complete high school and to make other educational transitions [14–16]. Most notably, a study by Fergusson and colleagues [17] provides results in line with social selection as they found that the increased risk of mental disorders among individuals without upper secondary credentials attenuated to non-significance when controlling for childhood adversity and adolescent mental health problems at 14 to 16 years of age.

One overlooked factor in the relationship between education and mental disorders is delayed educational progression. International reports indicate that at least 10 % of HS graduates in Denmark, Finland, Iceland, New Zealand, Norway and Portugal are 25 or older [18], suggesting that it is relatively common to spend more than the normative amount of time before completing high school (HS). Whether delayed HS completion is associated with higher symptom levels of anxiety and depression in adulthood and whether it influences the educational gradient is not known. Consequently, we aim to investigate whether delayed HS is associated with symptom level of adulthood anxiety and depression. We will also assess the association between educational attainment, and the combined effects of delayed HS completion and educational attainment on symptom levels of anxiety and depression in adulthood.

## Methods

### Sample

The sample is based upon the third wave of the Nord-Trøndelag Health Survey (HUNT 3), conducted in 2006. The HUNT studies are repeated cross sectional health surveys in which the entire adult population of the Nord-Trøndelag county has been invited to participate. About 60 000 out of the 105 000 invited individuals agreed to participate, yielding a response rate of 56 %. The geographical region where the study was conducted has a relatively homogenous population including less than 3 % non-Caucasians. The region is in many ways representative of the general Norwegian population, although it does not contain a larger city. The HUNT study, and the linking of the HUNT study with the registries has been approved by the Regional Committees for Medical and Health Research Ethics in Norway. Participants in this study have given written personal consents. A further description of the HUNT study is available elsewhere [19].

The data from the HUNT 3 survey was linked to the National Education Database in order to include detailed information about the educational background of the sample. Data files were de-identified by Statistics Norway and the identity of participants was thus unknown to the researcher at all times.

The current study has a prospective design using data from a total of 10 149 individuals participating in the HUNT 3 study. Participants younger than 30 years of age at the time of the HUNT 3 study were excluded, as these participants could still be in education in 2006. Participants older than 46 years of age were also excluded, as the data regarding initiation and graduation of HS were not considered reliable for these individuals. Individuals who had not completed HS within the HUNT 3 data collection (2006) were excluded from analyses presented in Table 2 (*N* = 7 968).

### Measures

Anxiety and depression were measured using the Hospital Anxiety and Depression Rating Scale (HADS), comprising 14 four-point Likert-scaled items with seven items relating to anxiety and depression, respectively. The questions aim to address the subjective experience of symptoms. Items in the scale are scored from 0 to 3 where a higher score indicate higher symptom frequencies. The HADS scale has been found to be a reliable measure of symptom severity in a general population [20]. We included all respondents who had responded to five or more of the anxiety and depression items respectively. Consequently, less than 2 % of respondents were excluded from the analyses due to missing response. We calculated a mean score of the items and multiplied this by 7. To check robustness we dichotomized anxiety and depression in line with the recommended cutoff for caseness (over 8 points) which has been found to give sensitivity and specificity in the range of 0.70 to 0.90 [20].

The variable referring to delayed completion of high school was based upon data from the National Educational Database. We constructed a dichotomous variable indicating whether the participant had completed high school within 5 years after starting (normative HS completion). The expected time to complete high school is 3 year and the 5 year limit was based on the Norwegian educational law granting students up to 5 years to complete high school [21]. Exceeding 5 years was thus considered a substantial delay. Additional analyses were done to determine the change in symptom levels associated with each additional year of delay in HS completion. A value of 0 represent completing HS within the expected 3 years, whereas a value of 1 indicate being 1 year delayed, a value of 2 indicating being 2 years delayed and so on. Being 6 years or more delayed was truncated into the value of 6.

Categories of educational attainment were constructed for Table 1 representing participants’ highest level of educational attainment in 2005. The levels include *elementary school*, *HS delayed*, *HS normative, bachelor level*, and *Master level, including postgraduate levels*. In order to avoid too few cases in some of the cells when testing for interactions, we collapsed *bachelor level* and those above into *higher education*. Individuals with high school as their highest level of educational attainment were labeled *lower education* in Table 2. The age variable was based upon participants’ age when the HUNT data was collected in 2006. This variable was categorized as 30 through 35 years of age, 36 through 40 years of age, and 41 years and older. Parental educational status was a combined measure indicating the educational level of the parent with the highest level of educational attainment. The categories were 1) less than high school, 2) high school or equivalent, or 3) higher educational attainment (any completed university or college education).

**Table 1:** Analysis of variance and mutually adjusted linear regression estimating differences in symptom load between educational categories and across demographic background variables

		Anxiety						Depression					
						Linear regression estimates						Linear regression estimates	
	N	Mean anxiety	sd	F-test	Multiple comparisons	b	95 % CI	Mean depression	sd	F-test	Multiple comparisons	b	95 % CI
Educational attainment													
1 Masters/PhD	539	3.75	2.87	***	4,5	ref		2.35	2.41	***	3,4,5	ref	
2 Bachelor level	2746	3.84	3.17		4,5	−0.07	(−0.38 to 0.23)	2.40	2.59		3,4,5	0.14	(−0.12 to 0.39)
3 HS normative	2878	3.88	3.48		4,5	0.09	(−0.22 to 0.40)	2.75	2.68		1,2,4	0.41	(0.15 to 0.67)
4 HS delayed	1862	4.54	3.09		1,2,3	0.72	(0.40 to 1.05)	3.17	2.85		1,2,3	0.76	(0.48 to 1.03)
5 Elementary school	2124	4.62	3.57		1,2,3	0.75	(0.42 to 1.07)	3.17	2.99		1,2,3	0.81	(0.54 to 1.08)
Gender													
Men	4116	3.83	3.04	***		ref		3.03	2.79	***		ref	
Women	6058	4.35	3.44			0.57	(0.44 to 0.70)	2.64	2.73			−0.32	(−0.44 to −0.21)
Parental educational status													
Higher education	1492	3.81	3.01	***		ref		2.64	2.68	***		ref	
HS or equivalent	1099	4.04	3.20			0.11	(−0.15 to 0.36)	2.57	2.61			−0.03	(−0.25 to 0.18)
Less than HS	7444	4.20	3.35			0.17	(−0.02 to 0.36)	2.85	2.78			0.04	(−0.12 to 0.20)
Age in 2006													
30 to 35	2542	4.15	3.19			ref		2.57	2.6	***		ref	
36 to 40	3169	4.08	3.26			−0.13	(−0.30 to 0.05)	2.76	2.74			0.13	(−0.01 to 0.28)
41 +	4463	4.17	3.38			−0.15	(−0.31 to 0.02)	2.96	2.86			0.24	(0.10 to 0.38)
Total	10149

**Table 2:** Predicted mean scores and pairwise comparisons between the different combinations of educational attainment and normative/delayed HS completion

			Anxiety symptoms			Depression symptoms	
	N	Mean	95 % CI	Sig different from	Mean	95 % CI	Sig different from
Higher educational attainment and							
1 Normative HS	2733	3.75	(3.63 to 3.87)	4	2.42	(2.32 to 2.52)	3,4
2 Delayed HS	399	3.87	(3.57 to 4.18)	4	2.56	(2.30 to 2.81)	4
Lower educational attainment and							
3 Normative HS	2679	3.92	(3.80 to 4.04)	4	2.74	(2.64 to 2.84)	1,4
4 Delayed HS	1705	4.55	(4.40 to 4.70)	1,2,3	3.08	(2.96 to 3.21)	1,2,3

### Statistical analysis

One way ANOVA were used to test for differences in means across categories of educational attainment and across covariates. Bonferroni corrections were used to correct for multiple testing for the ANOVA models. Linear regression models were used to estimate associations and 95 % CI between symptom levels of anxiety and depression and the predictors. Interactions between delayed/normative HS completion and educational attainment were tested by including a multiplicative term (delayed HS completion*low educational attainment) in a multiple linear regression model with the main effects and dummy-coded covariates (age, gender, and parental educational attainment). We also used the F-test statistic to assess the improvement in overall model fit when including the interaction term. The regression model including the interaction term was used to calculate predicted mean scores for each combination of the two variables. Pairwise comparisons were conducted to assess the extent to which different combinations of educational attainment and delayed/normative HS completion were associated with different levels of anxiety or depressive symptoms. Stata version SE 13.0 was used for all statistical analyses.

## Results

### Distribution of anxiety and depression symptoms across levels of educational attainment and normative/delayed HS completion

Our findings indicated an educational gradient where lower educational attainment was associated with higher symptom levels of anxiety and depression, and that delayed completion of HS was associated with relatively higher symptom levels of both, as compared to normative HS completion (Table 1). Symptom levels of both anxiety and depression were significantly higher among individuals with elementary school and high school with delayed completion relative to individuals with bachelor or master/PhD level education.

The results indicated gender-specific patterns, as women were found to have higher symptom levels of anxiety, while men were found to have higher symptom levels of depression. The F-test statistic indicated significant differences in symptom levels across categories of parental educational attainment, but the differences were no longer significant in the multiple regression models. Age was found to be associated with symptom levels of depression with older participants reporting higher symptom levels. No such pattern was evident for anxiety symptoms.

Additional analysis (table not shown) indicated that the prevalence of delayed HS completion was highest among individuals with HS as their highest level of educational attainment (39.4 %), and lower for individuals with higher levels of educational attainment (14.7 % bachelor level, 5.3 % for master/PhD). Delayed HS completion was more common among women (31.3 %) than men (26.7 %). Crude regression models suggested that these differences in educational level and gender were statistically significant (*P* < .05). A total of 28.6 % of the sample were delayed in their HS completion.

### Interactions between educational attainment and delayed HS completion in predicting symptom levels of anxiety and depression

We found a significant interaction between educational attainment and normative/delayed completion of HS when predicting anxiety symptoms (*P* < 0.05) and a significant F-test statistic when including the interaction term (*P* < 0.05). For depression, neither the interaction term nor the F-test was found to be statistically significant (*P* < 0.05) (table not shown). This indicates that the association between educational attainment and anxiety differs between individuals who have a normative HS completion and individuals who do not. It also suggests that the association between educational attainment and depression is consistent across individuals whether they completed HS in a normative or a delayed timeframe.

This interaction is highlighted by Table 2 showing comparisons of predicted means across the different combinations of educational attainment and normative/delayed HS completion. For anxiety, we found significantly elevated symptom levels among individuals with a combination of lower educational attainment and delayed HS completion relative to any other combination of educational attainment and delayed/normative HS completion. Anxiety levels were not found to differ between the other combinations of educational attainment and delayed/normative HS completion. While there was no significant interaction in predicting depressive symptoms, we still found a similar pattern indicating that the combination of having lower educational attainment and delayed HS completion was associated with elevated symptom levels compared to all other groups. Individuals with lower educational attainment and normative HS completion were also found to have significantly higher symptom levels than those with higher educational attainment and normative HS completion.

### Dose–response associations between delayed HS completion and symptoms of anxiety and depression

The importance of the number of years delayed for the risk of high symptom levels of depression and anxiety was estimated in linear regression models using delayed completion of HS as a continuous variable. The results from these analyses suggest that each year of delayed completion of HS was associated with a .10 point increase in depression symptoms (95 % CI (.07 to .12)) and with an increase of .12 points per year for anxiety symptoms (95 % CI (.09 to .16)). Interactions between years of delay and higher educational attainment were not found to be statistically significant.

### Robustness checks

Replication of the main findings with logistic regression models scores confirmed the main findings from the linear models. Both logistic and linear regression models were also replicated within a sub-sample (*N* = 7194) excluding all participants who reported having had *long lasting disease, injury or disorder of physical or mental nature*, or participants who had *sought help for mental problems*. These results also confirmed the main findings reported with one exception. The group with higher educational attainment and delayed HS completion was found to have significantly higher OR for high symptom levels of depression (OR = 1.55 (1.00 to 2.38)) relative to the reference group.

## Discussion

The main findings of the current study suggest a dose–response relationship indicating that each additional year of delayed HS completion is associated with increased symptom levels of both anxiety and depression. For anxiety, we found that being substantially delayed in completing HS was associated with higher anxiety symptoms for those with lower, but not for those with higher levels of educational attainment. For depression, the association between delayed HS completion and symptom level was found to be consistent across levels of educational attainment.

Our findings concur with those of previous studies in identifying a higher symptom level of both anxiety and depression among individuals with lower educational attainment [1, 4]. A key finding is that the educational gradient in symptom levels of anxiety and depression is not only dependent upon the level of educational attainment, but also upon the time it takes to complete HS. This is supported by the results indicating that symptom levels for both anxiety and depression did not differ between individuals with elementary school as their highest level of educational attainment and those with delayed completion of high school. We also found that individuals with HS as their highest level of educational attainment had lower symptom levels of both depression and anxiety if they completed within a normative timeframe relative to those who spent more than 5 years completing HS.

The association between delayed HS completion and adulthood symptoms of anxiety and depression has, to the authors’ knowledge, not been described in previous studies. Little is therefore known about the mechanisms explaining the increase in symptoms for both anxiety and depression in adulthood among individuals with delayed high school completion. We propose social selection and social causation as two potentially complimentary explanations for this association. A social selection explanation would suggest that individuals with an early onset of CMD or symptoms thereof, will find it more difficult to complete HS. This would correspond well with studies indicating that high symptom levels [22, 23] or internalizing disorders [14, 24] during adolescence are associated with non-completion of HS within the early twenties. A social causation explanation on the other hand, would suggest that the increased symptom levels among individuals with delayed HS completion may be caused by stress related to failing to follow a normative educational progression, or simply by a lack of positive experiences in the school setting. Complementary to this, successful completion of high school could boost mastery and self-efficacy, factors associated with positive mental health. To our knowledge, no studies have investigated the merits of these potential mechanisms regarding delayed completion of HS.

A statistical interaction indicated that delayed HS completion was associated with anxiety symptoms among individuals with lower, but not among those with higher levels of educational attainment. A selection explanation of these findings may suggest that individuals with an early onset of anxiety or a predisposition for developing symptoms of anxiety may be more at risk of being delayed in HS, or being less likely to later successfully complete higher education. While there is some support of non-completion of HS by early twenties associated with early onset disorders [14, 22–24], only one [15] of three known studies have found that early onset disorders reduce the odds for completing higher educational attainment. The two other studies did not find significant associations between higher educational attainment and mood disorders or anxiety disorders [16, 25]. It is important to note that these results are not entirely comparable to ours as they only include individuals who were admitted to college or university, whereas the current results also include individuals who never attended higher education. A social causation mechanism explanation for these findings could suggest that the combination of delayed HS completion and no higher educational attainment may lead to a particularly difficult life situation with regard to employment, or with other kinds of social stress which in turn could lead to increased symptoms of anxiety.

For depression, we found no interactions, indicating that the associations between educational attainment and normative/delayed HS completion were additive and did not vary across different levels of each other. The absence of interactions illustrates a potentially important difference between the predictors of case-level anxiety and case-level depression. The reasons for these differences should be investigated in studies with repeated measures of CMD throughout adolescence and early adulthood, but is not possible to determine with our current dataset.

A key contribution of the current study is that the results shed light upon delayed HS completion as a largely ignored factor which complicates the much studied relationship between educational attainment and mental health. Several important questions remain unanswered and we encourage future studies to investigate the role of delayed HS completion and mental health in more depth. First, we know little about reasons for delayed HS completion and the extent to which delays caused by reasons other than mental health impairments are associated with mental health outcomes. Second, we do not know the relative contributions of social causation and selection mechanisms in explaining the associations reported herein. Finally, we also do not know whether our results are robust across different countries, school systems, and types of mental disorders.

Limitations of this study include exclusion of population subgroups. The health survey which provided information about common mental disorders did not include institutionalized individuals, minorities with poor Norwegian language skills, and those with severe mental illnesses who are unlikely to respond to surveys. The use of self-reported symptom scales rather than clinical interviews may also be considered a limitation. However, self-report symptom scales also have some advantages as they may be used to capture the importance of sub-clinical symptom levels and how the symptom continuum is associated with other variables.

Strengths of our study include a large sample providing sufficient statistical power, well validated instruments [20, 26], and the use of multiple data-sources.

This study has several implications. For public health and mental health prevention, we believe that screening for case-level anxiety and case-level depression among individuals with delayed HS progression could be beneficial as this group has significantly higher odds of both case-level anxiety and case-level depression relative to those who follow a normative progression. Early identification of individuals at high risk for CMD may in turn be useful for targeted interventions providing counseling with regard to education, employment, or mental health related issues. Our findings also have implications for epidemiological studies as estimates of risk associated with different levels of educational attainment may be misleading when ignoring whether individuals have followed a normative or delayed progression in their studies.

## Conclusions

The current study has found that delayed HS completion is associated with increased levels of both anxiety and depression symptoms in adulthood. Each additional year of delay in completing HS was found to be associated with higher symptom levels. The association between delayed HS completion and anxiety symptoms varied across levels of educational attainment as there was no significant association between delayed HS completion and anxiety symptoms for individuals with higher educational attainment. The mechanism explaining why delayed HS completion is associated with adulthood anxiety and depression is not known and should be investigated in future studies.
